# Supplementary material for: HuD regulates apoptosis in N2a cells by regulating Msi2 expression
Source: PLoS One. 2024 Dec 16;19(12):e0315535. doi: 10.1371/journal.pone.0315535 (PMC11649143; doi:10.1371/journal.pone.0315535)
Supplement: S1 Fig — Control siRNA and HuDsi RNAs were transfected in N2a cells and the RNA levels of Msi2 RNA were examined by Real-Time PCR A) Graph shows the decrease in the Msi2 RNA levels. (PPTX) [file pone.0315535.s001.pptx]

## Slide 1
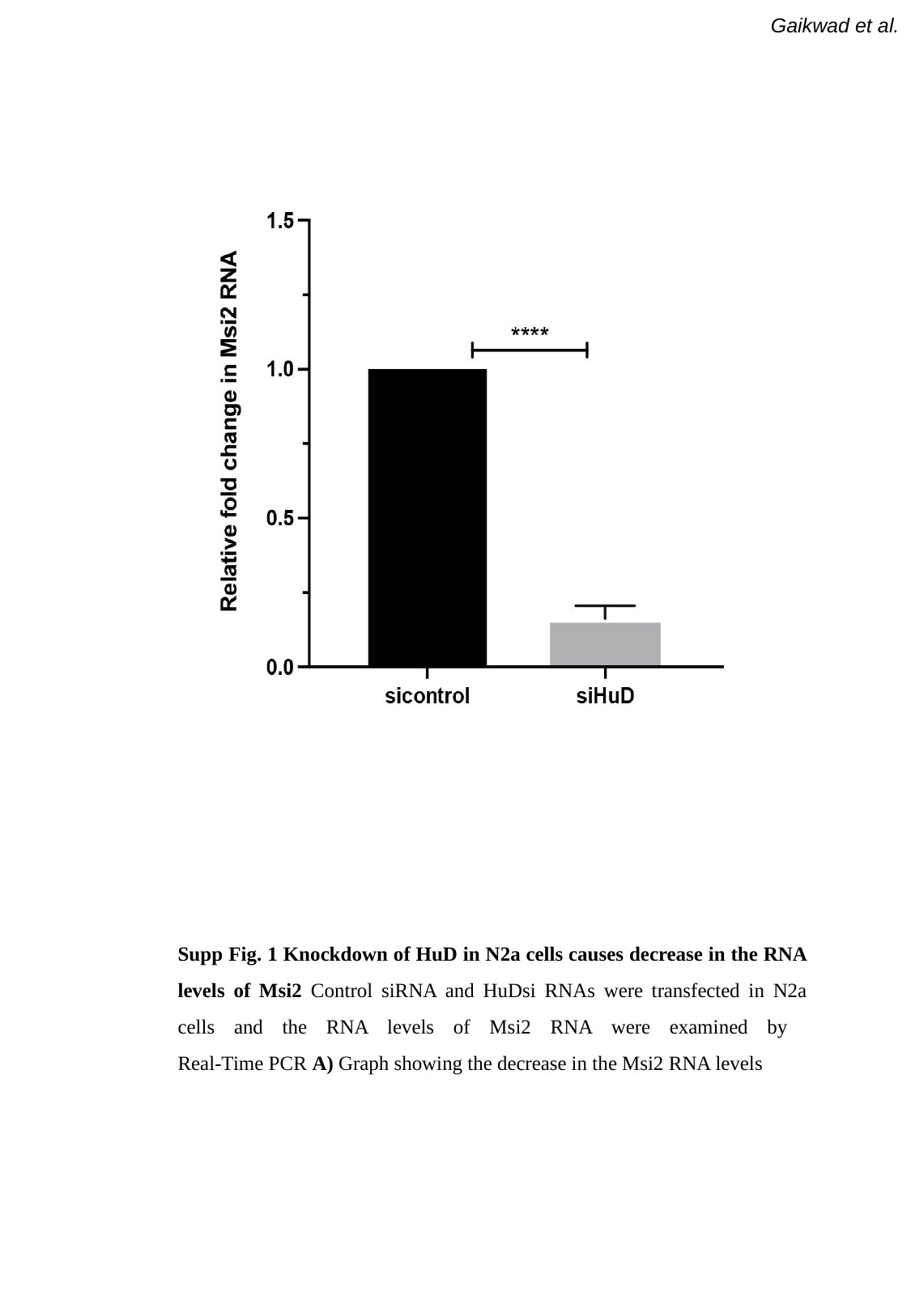

Gaikwad et al.
Supp Fig. 1 Knockdown of HuD in N2a cells causes decrease in the RNA levels of Msi2 Control siRNA and HuDsi RNAs were transfected in N2a cells and the RNA levels of Msi2 RNA were examined by
Real-Time PCR A) Graph showing the decrease in the Msi2 RNA levels
