# Supplementary material for: HuD regulates apoptosis in N2a cells by regulating Msi2 expression
Source: PLoS One. 2024 Dec 16;19(12):e0315535. doi: 10.1371/journal.pone.0315535 (PMC11649143; doi:10.1371/journal.pone.0315535)
Supplement: S2 Fig — peGFP.C1 EV and peGFP.C1 Msi2 constructs were overexpressed in N2a cells and the protein levels of APAF1 was analyzed by western blotting. (A) Western blot showing protein levels of APAF1 upon Msi2 overexpression. (B) Bar graph represents the relative quantitation of the Msi2 and APAF protein normalized to tubulin levels from three independent western blots. (PPTX) [file pone.0315535.s002.pptx]

## Slide 1
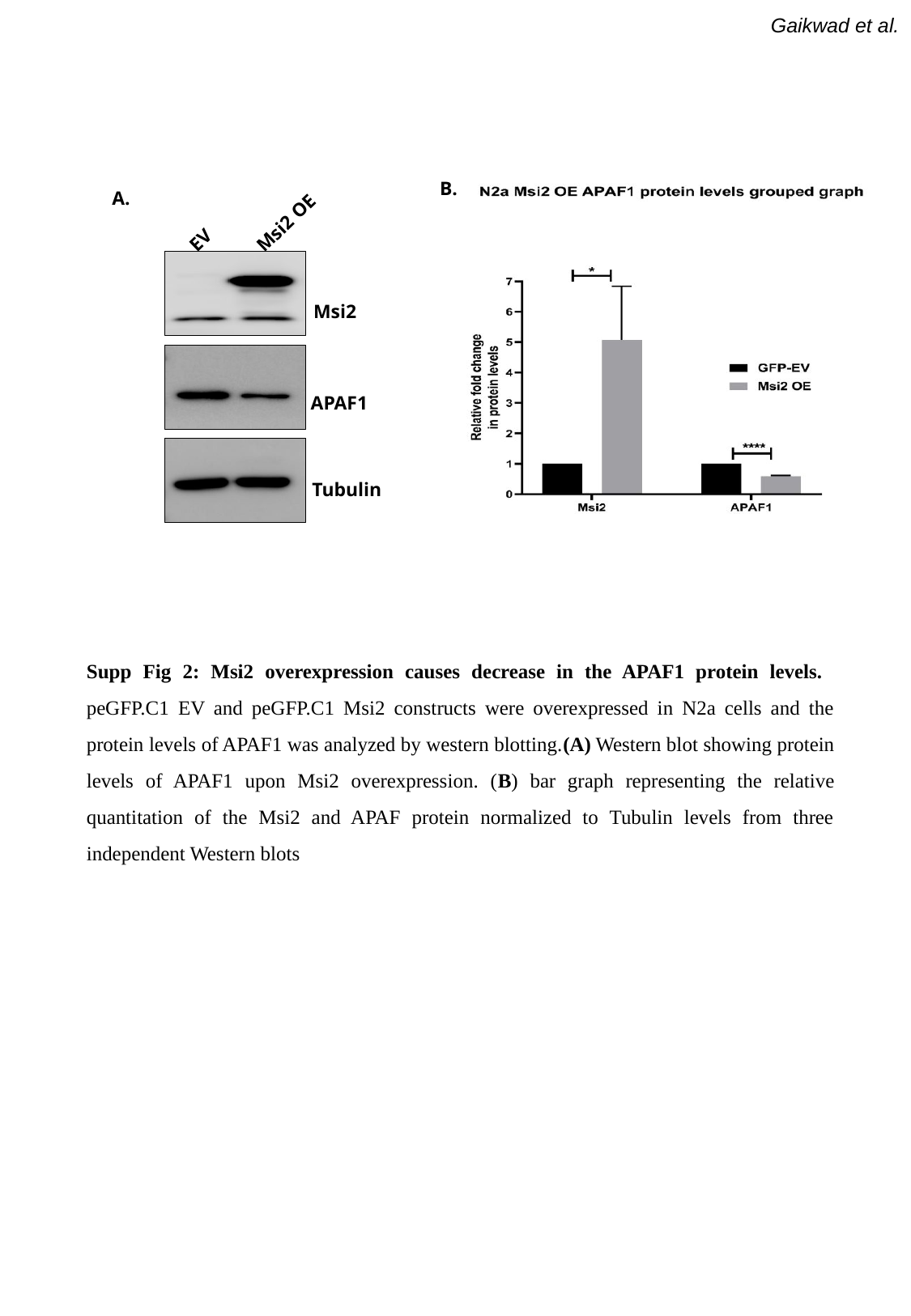

Gaikwad et al.
B.
A.
EV
Msi2 OE
Msi2
APAF1
Tubulin
Supp Fig 2: Msi2 overexpression causes decrease in the APAF1 protein levels. peGFP.C1 EV and peGFP.C1 Msi2 constructs were overexpressed in N2a cells and the protein levels of APAF1 was analyzed by western blotting.(A) Western blot showing protein levels of APAF1 upon Msi2 overexpression. (B) bar graph representing the relative quantitation of the Msi2 and APAF protein normalized to Tubulin levels from three independent Western blots
