# Supplementary material for: HuD regulates apoptosis in N2a cells by regulating Msi2 expression
Source: PLoS One. 2024 Dec 16;19(12):e0315535. doi: 10.1371/journal.pone.0315535 (PMC11649143; doi:10.1371/journal.pone.0315535)
Supplement: S3 Fig — Double knockdown of HuD and Msi2 was carried out in N2a cells using siRNA, the protein levels of APAF1 (A) and ratio of Bax/Bcl2 (B) were analyzed by western blotting. Western blot shows increase in the protein levels of APAF1 upon HuD and Msi2 double knockdown and its graphical representation (right panel). (PPTX) [file pone.0315535.s003.pptx]

## Slide 1
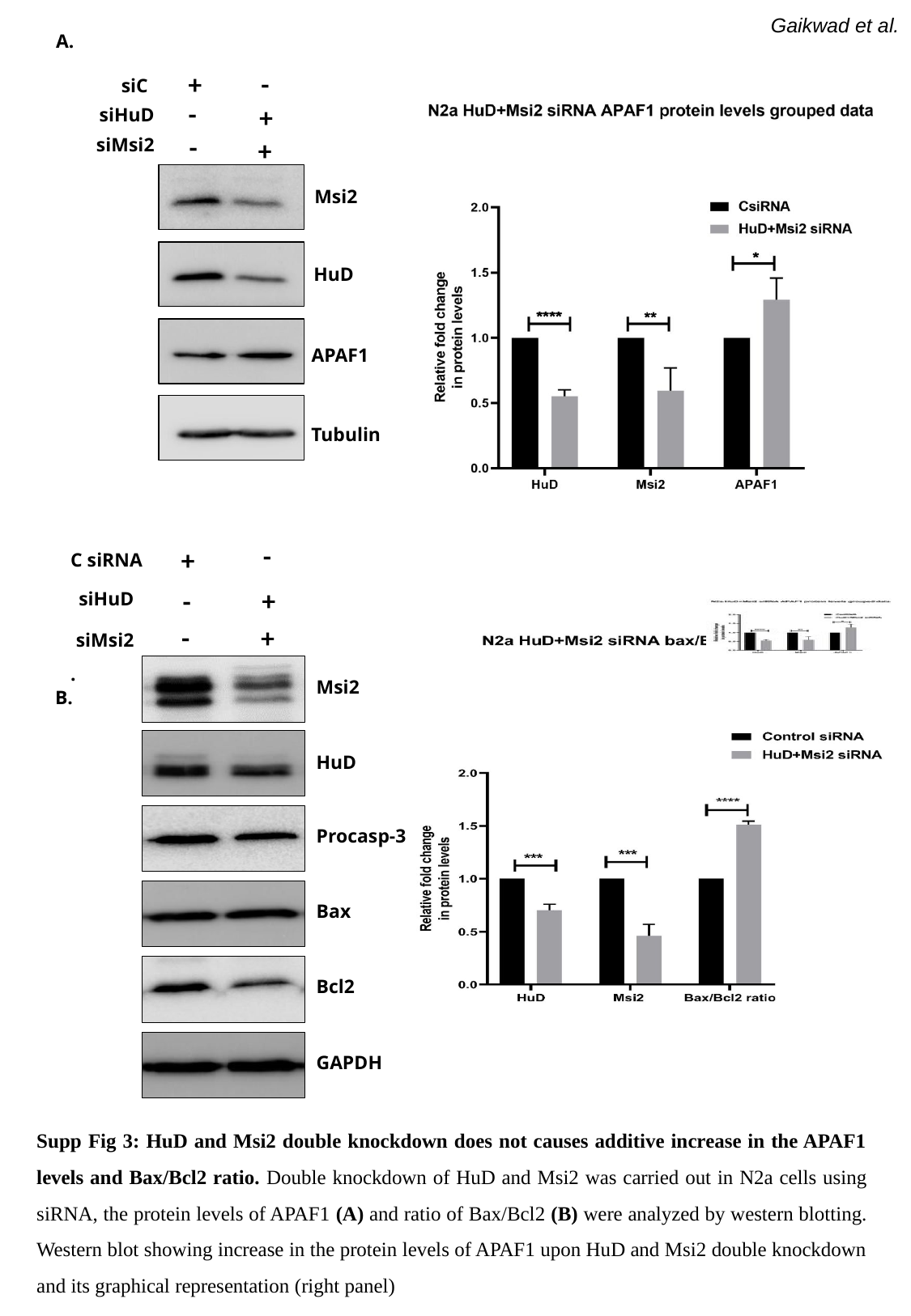

Gaikwad et al.
A.
+
-
siC
-
+
siHuD
-
siMsi2
+
Msi2
HuD
APAF1
Tubulin
-
+
C siRNA
+
-
-
+
Msi2
HuD
Procasp-3
Bax
Bcl2
GAPDH
siHuD
siMsi2
.
B.
Supp Fig 3: HuD and Msi2 double knockdown does not causes additive increase in the APAF1 levels and Bax/Bcl2 ratio. Double knockdown of HuD and Msi2 was carried out in N2a cells using siRNA, the protein levels of APAF1 (A) and ratio of Bax/Bcl2 (B) were analyzed by western blotting. Western blot showing increase in the protein levels of APAF1 upon HuD and Msi2 double knockdown and its graphical representation (right panel)
