# Supplementary material for: HuD regulates apoptosis in N2a cells by regulating Msi2 expression
Source: PLoS One. 2024 Dec 16;19(12):e0315535. doi: 10.1371/journal.pone.0315535 (PMC11649143; doi:10.1371/journal.pone.0315535)
Supplement: S4 Fig — HuD and Msi2 were sequentially overexpressed in N2a cells and the Bax/Bcl2 ration was examined by western blotting, A) Western blot showing decrease in the Bax/Bcl2 ration upon sequential overexpression of Hud and Msi2. B) Graphical representation of the same. (PPTX) [file pone.0315535.s004.pptx]

## Slide 1
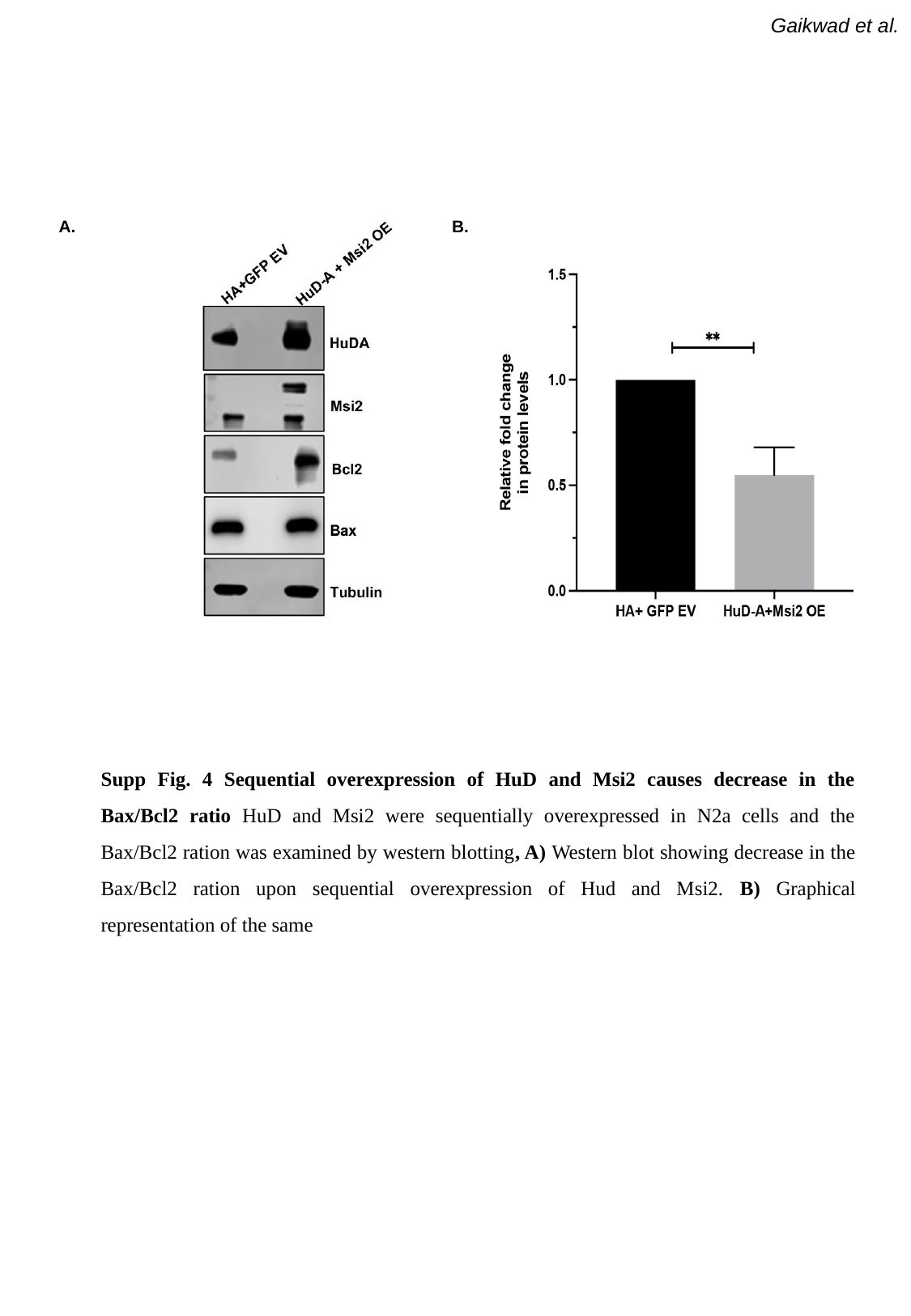

Gaikwad et al.
A.
B.
Supp Fig. 4 Sequential overexpression of HuD and Msi2 causes decrease in the Bax/Bcl2 ratio HuD and Msi2 were sequentially overexpressed in N2a cells and the Bax/Bcl2 ration was examined by western blotting, A) Western blot showing decrease in the Bax/Bcl2 ration upon sequential overexpression of Hud and Msi2. B) Graphical representation of the same
