# Supplementary material for: HuD regulates apoptosis in N2a cells by regulating Msi2 expression
Source: PLoS One. 2024 Dec 16;19(12):e0315535. doi: 10.1371/journal.pone.0315535 (PMC11649143; doi:10.1371/journal.pone.0315535)
Supplement: S1 Raw image — The specific figure number is as indicated. (PDF) [file pone.0315535.s005.pdf]

Fig: 1.A.

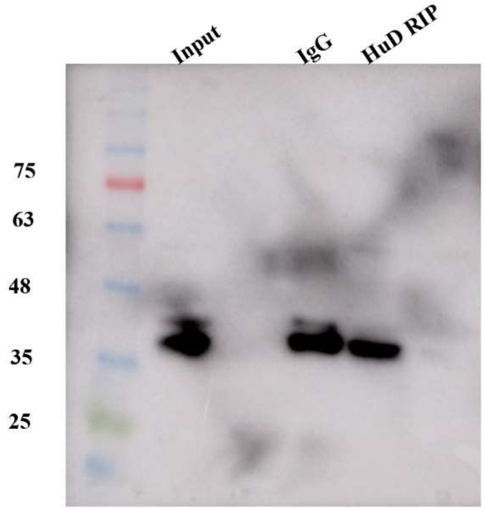
Input
IgG
HuD RIP
75
63
48
35
25

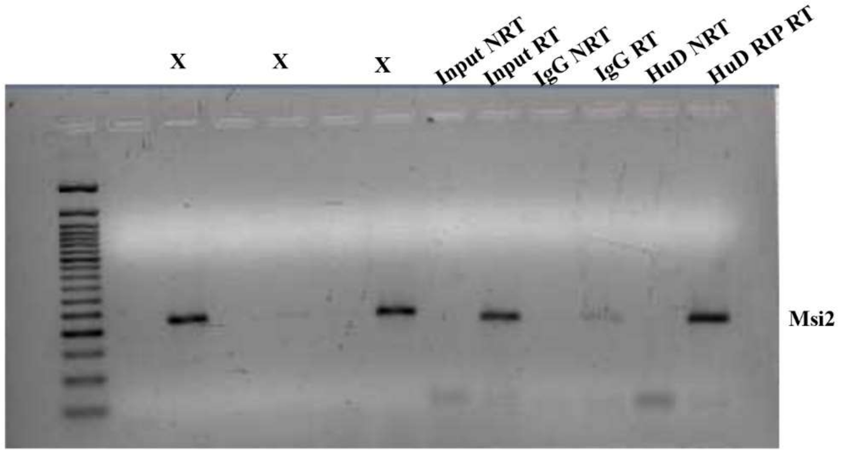
X
X
X
Input NRT
Input RT
IgG NRT
IgG RT
HuD NRT
HuD RIP RT
Msi2

Fig: 1.C.

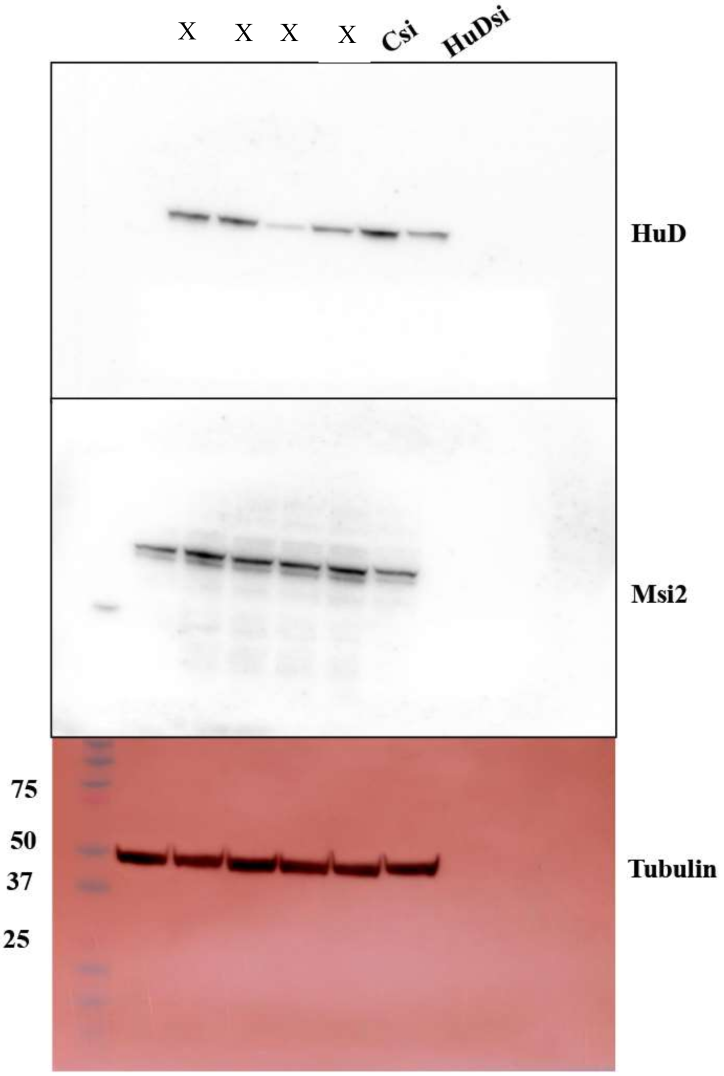

Fig: 2.A.

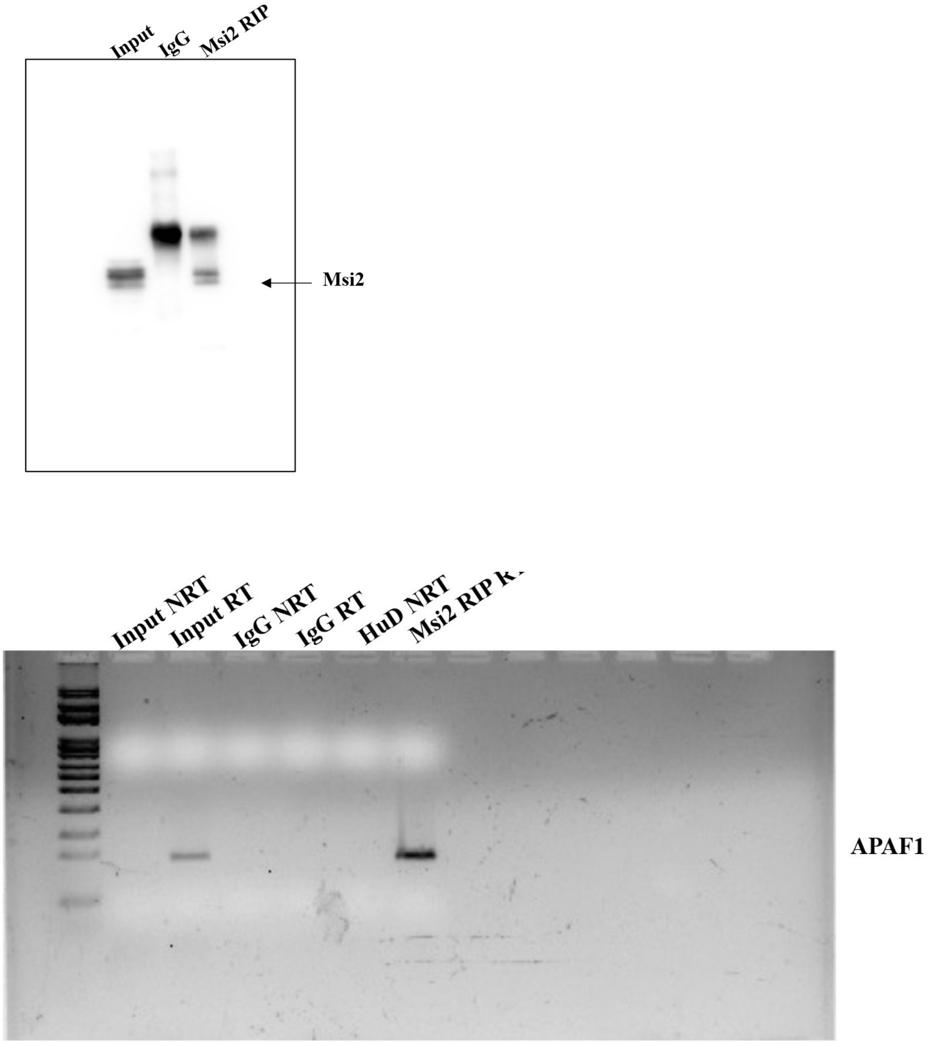
Input
IgG
Msi2 RIP
Msi2
Input NRT
Input RT
IgG NRT
IgG RT
HuD NRT
Msi2 RIP
APAF1

Fig: 2.C.

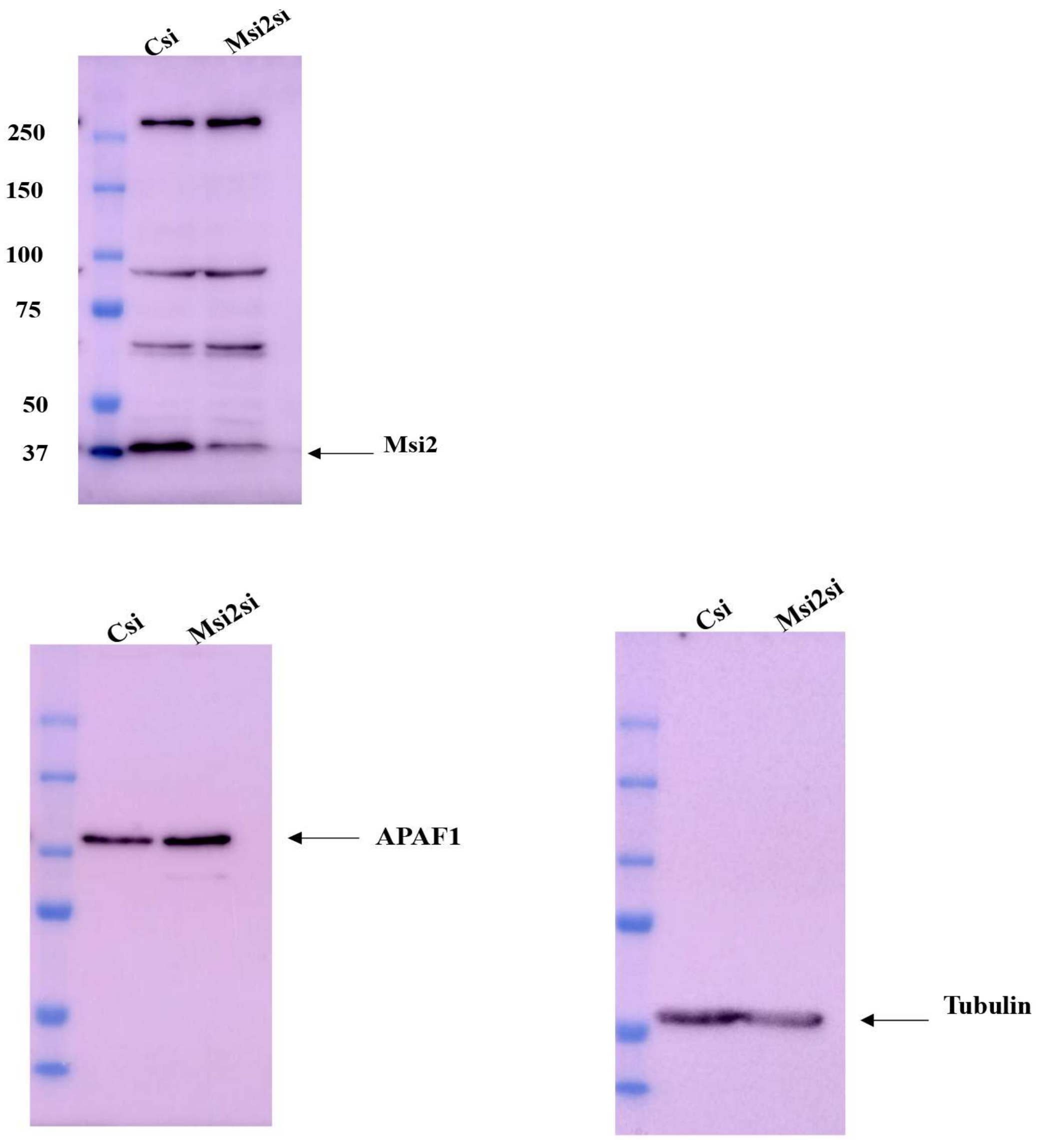

Csi
Msi2si
250
150
100
75
50
37
Msi2
Csi
Msi2si
APAF1
Csi
Msi2si
Tubulin

Fig: 3.D.

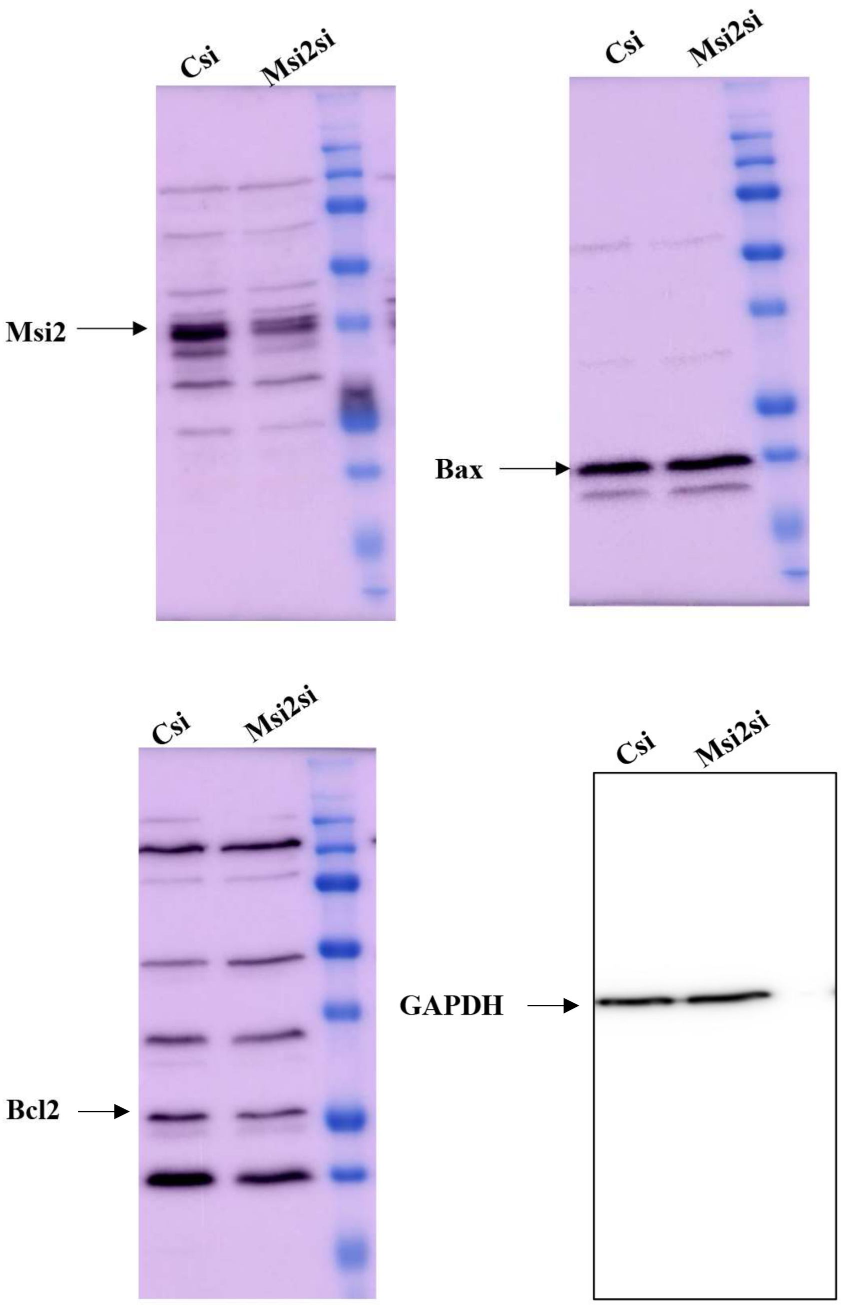
Csi
Msi2si
Msi2
Csi
Msi2si
Bax
Csi
Msi2si
Bcl2
Csi
Msi2si
GAPDH

Fig: 4.D.

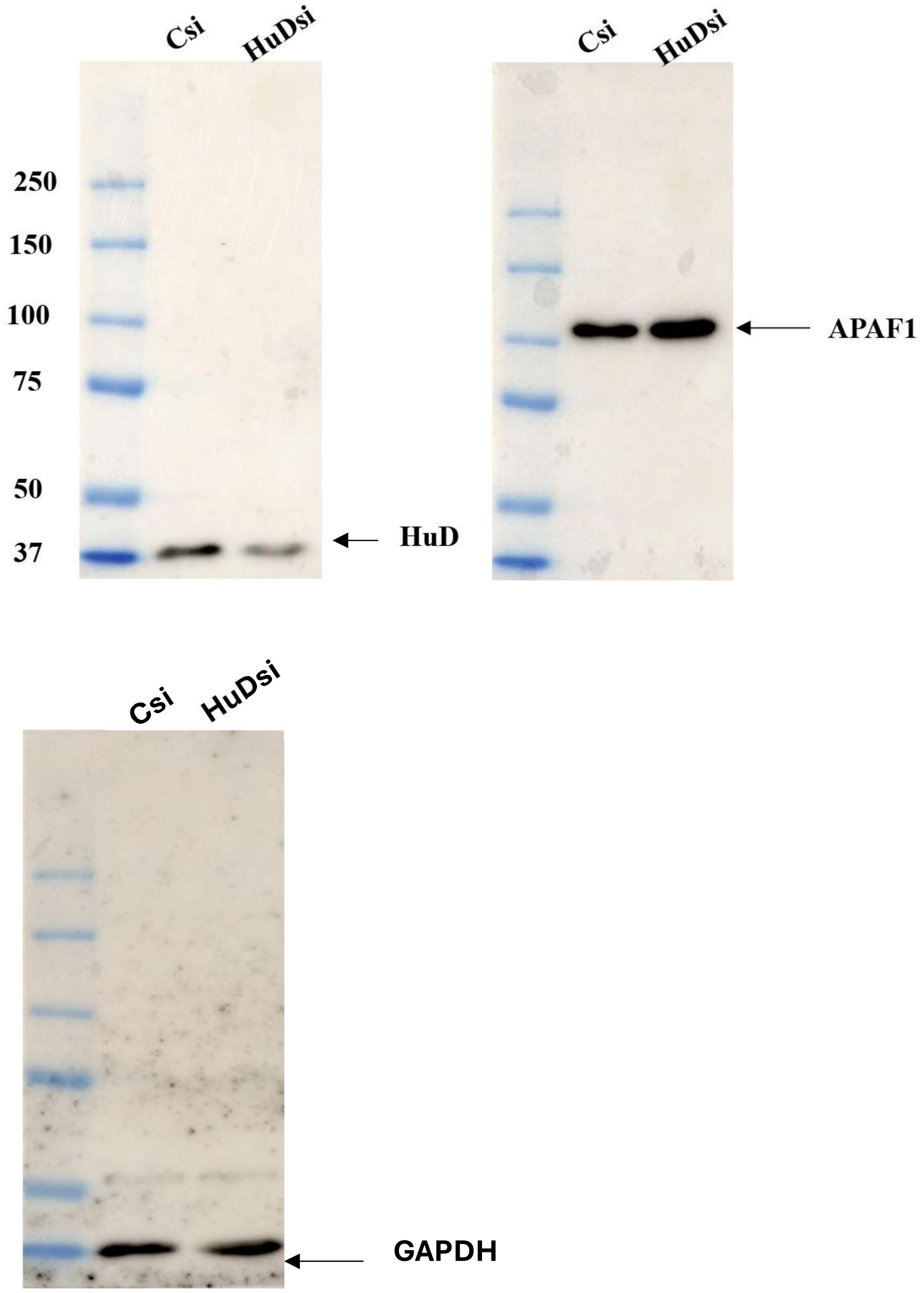
Csi
HuDsi
250
150
100
75
50
37
HuD
Csi
HuDsi
APAF1
Csi
HuDsi
GAPDH

Fig: 4 E

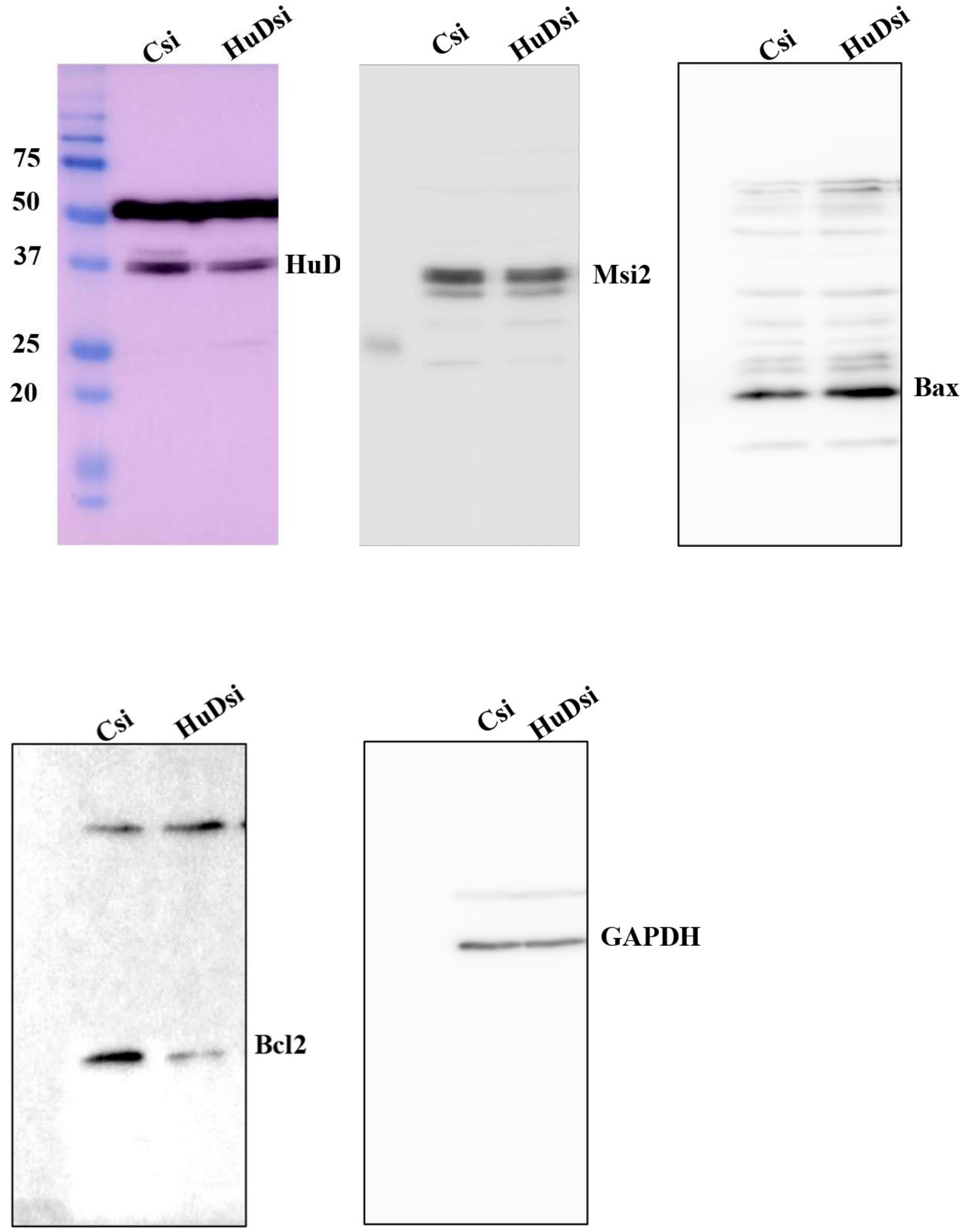
Csi
HuDsi
75
50
37
25
20
HuD
Csi
HuDsi
Msi2
Csi
HuDsi
Bax
Csi
HuDsi
Bcl2
Csi
HuDsi
GAPDH

Fig: 5 D

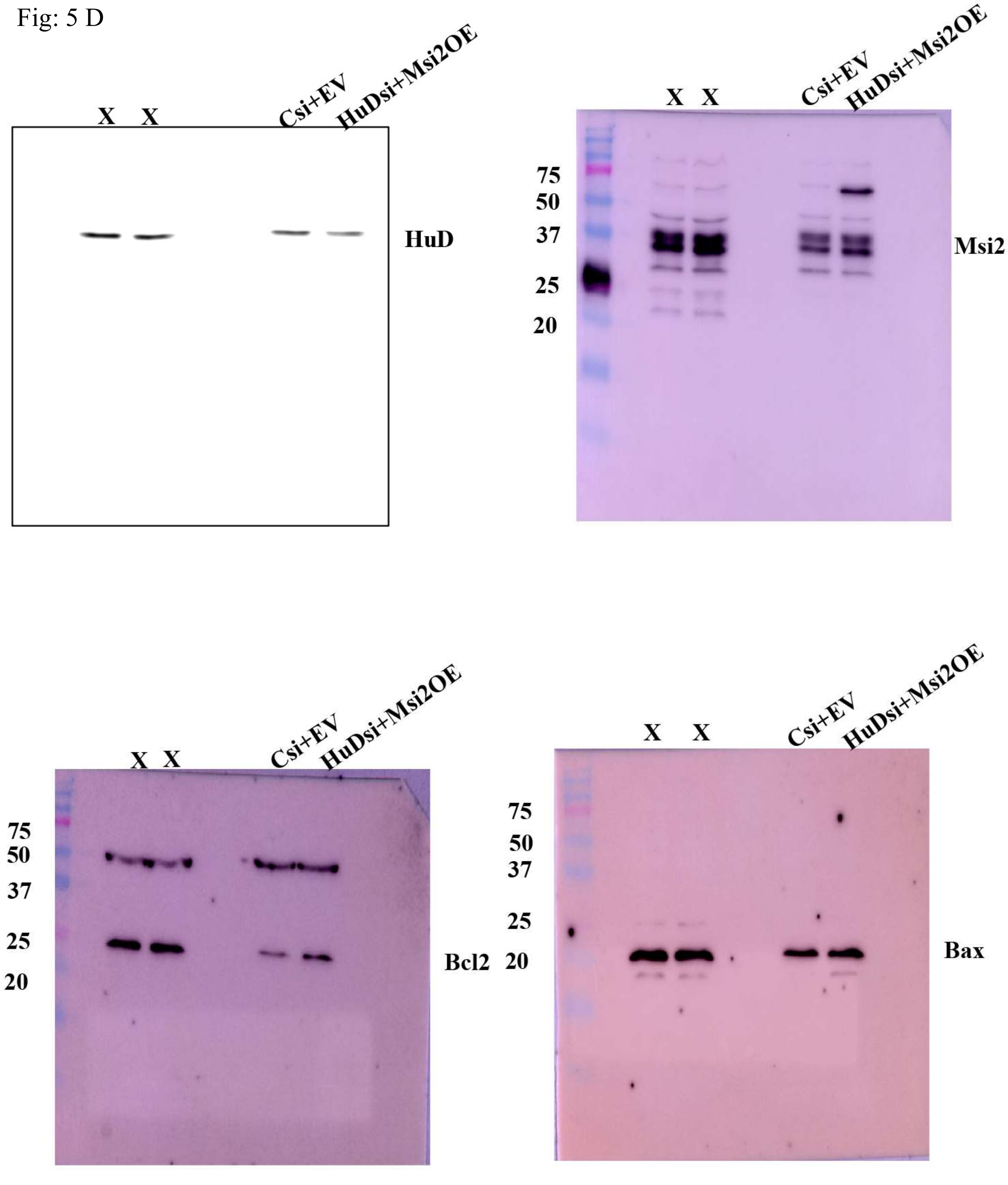

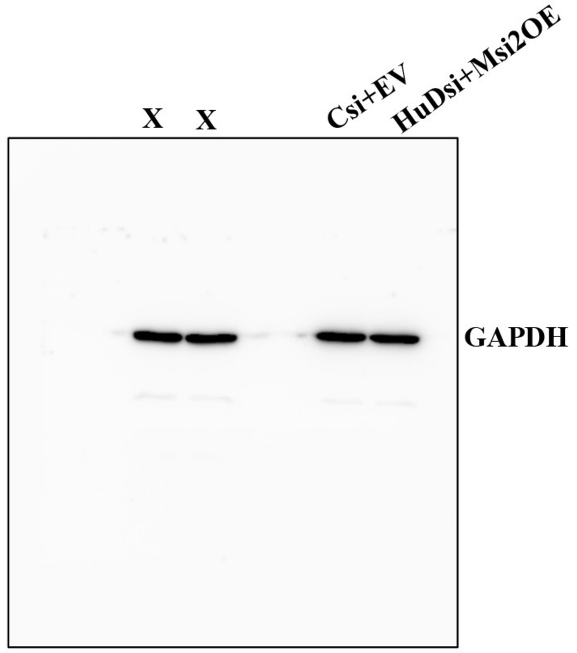

X
X
Csi+EV
HuDsi+Msi2OE
GAPDH

Fig: 5 D

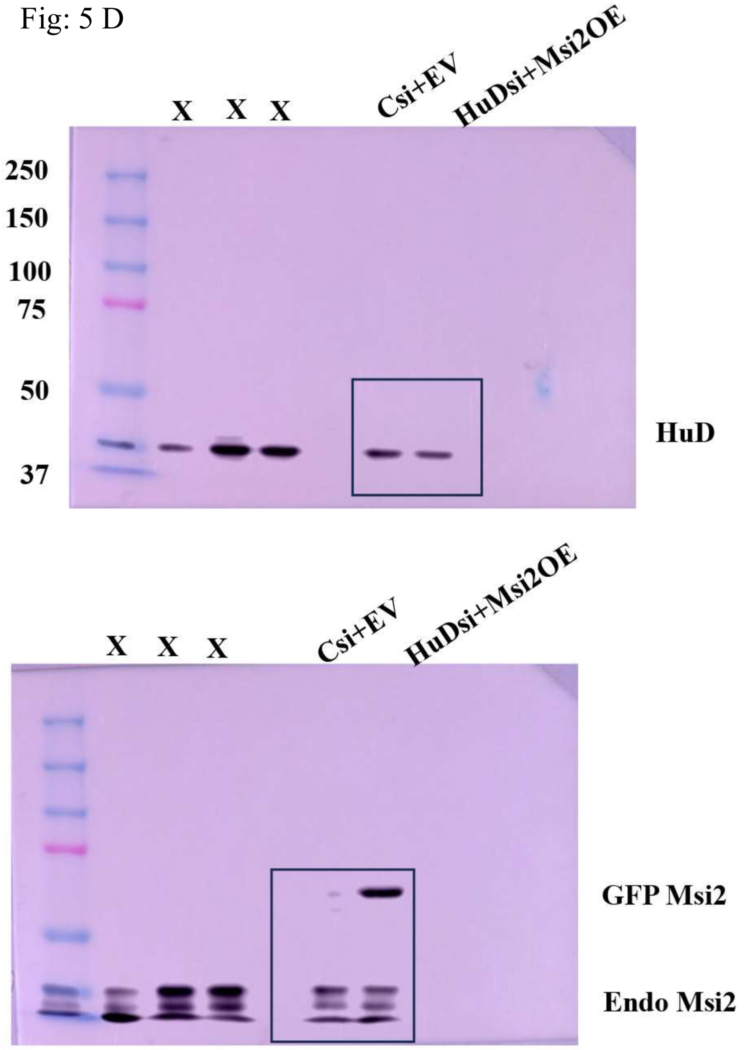
X
X
X
Csi+EV
HuDsi+Msi2OE
250
150
100
75
50
37
HuD
X
X
X
Csi+EV
HuDsi+Msi2OE
GFP Msi2
Endo Msi2

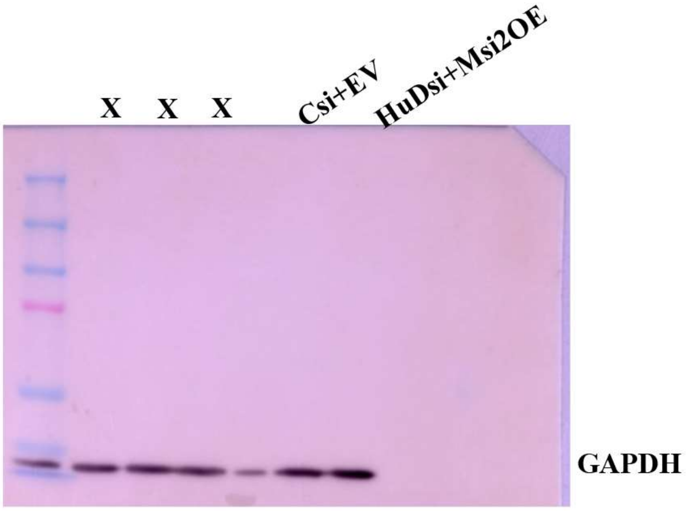
X
X
X
Csi+EV
HuDsi+Msi2OE
GAPDH

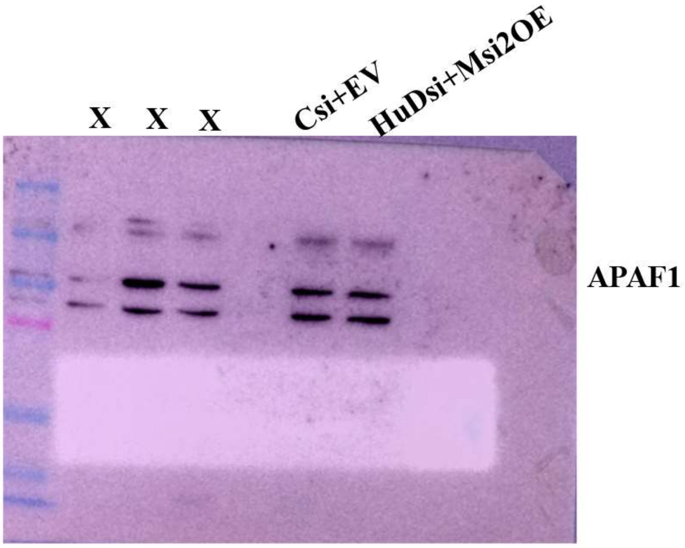
X
X
X
Csi+EV
HuDsi+Msi2OE
APAF1

Supp. Fig: S2 A

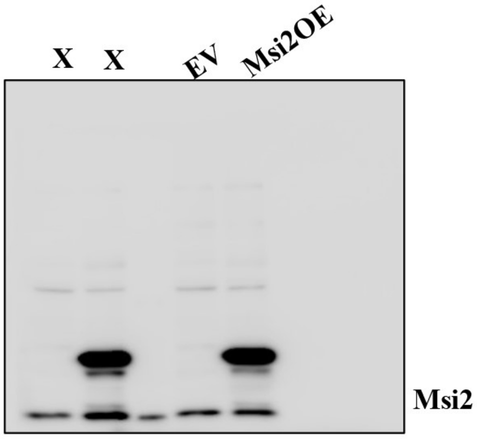
X
X
EV
Msi2OE
Msi2

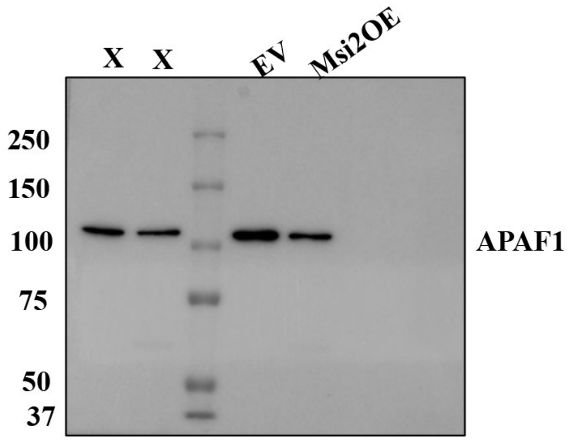
X
X
EV
Msi2OE
250
150
100
75
50
37
APAF1

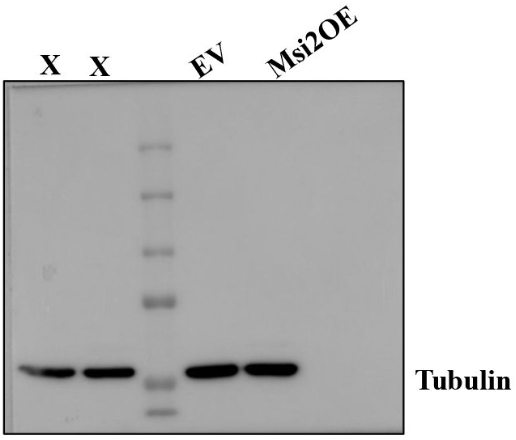
X
X
EV
Msi2OE
Tubulin

Supp. Fig. S3 A

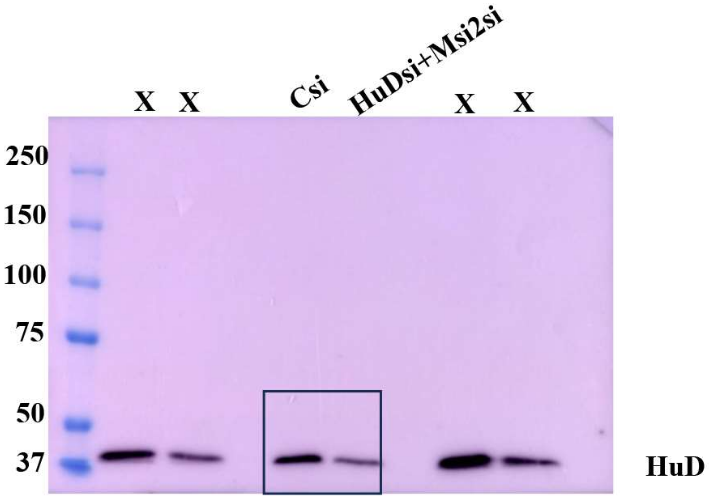
X X Csi HuDsi+Msi2si X X
250
150
100
75
50
37
HuD

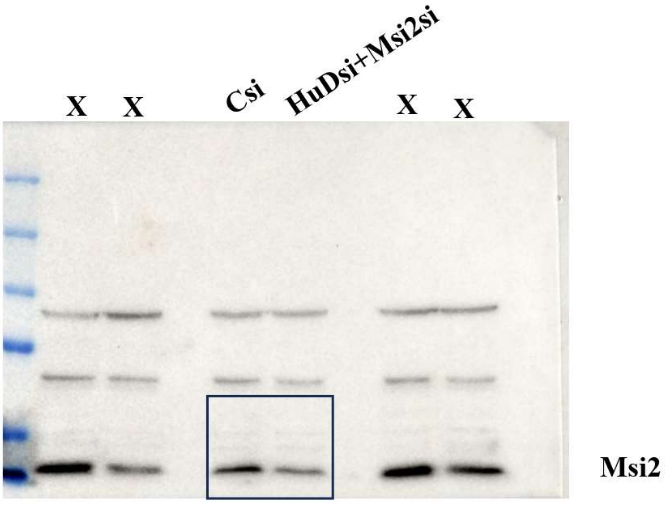
X X Csi HuDsi+Msi2si X X
Msi2

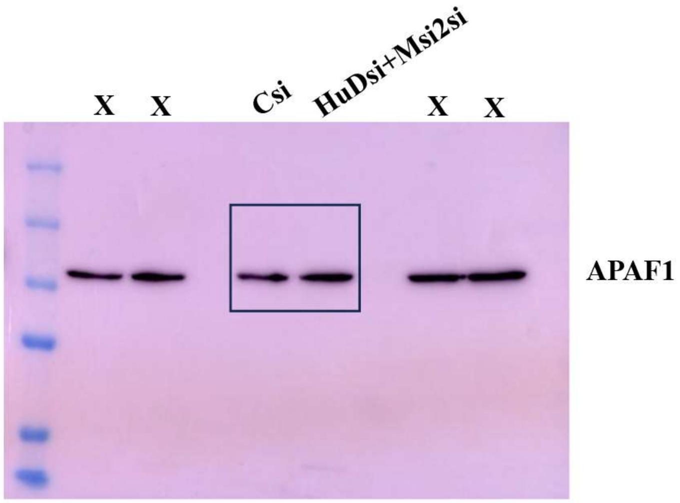
X
X
Csi
HuDsi+Msi2si
X
X
APAF1

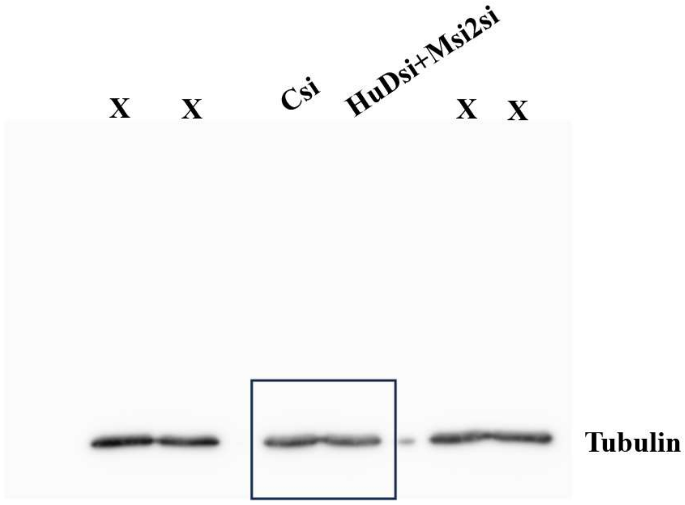
X
X
Csi
HuDsi+Msi2si
X
X
Tubulin

Supp. Fig. S3B
C

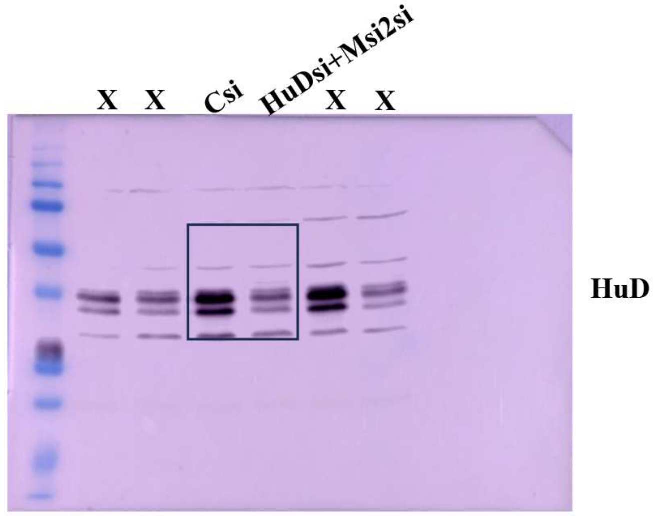

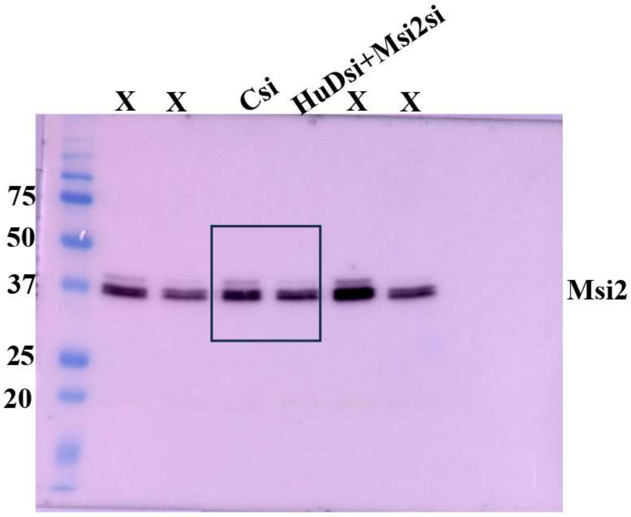

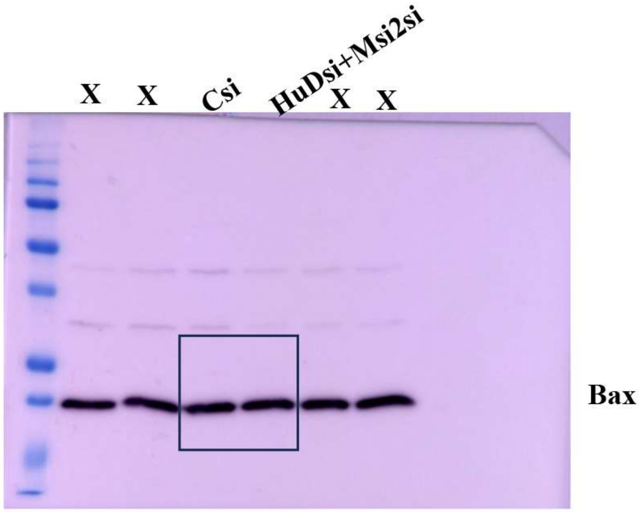

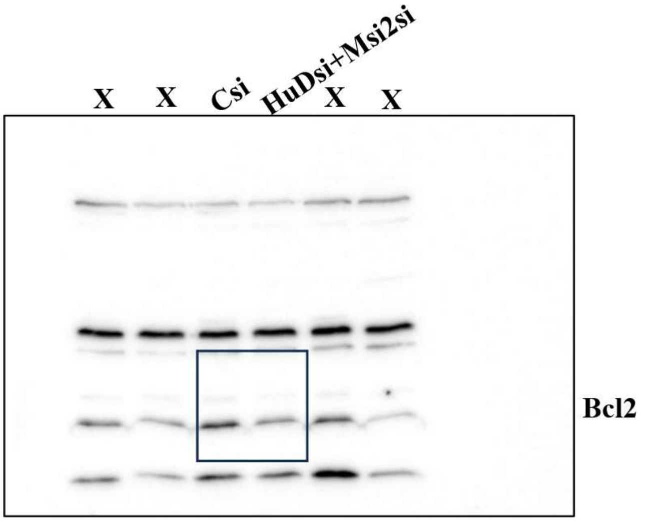
X
X
Csi
HuDsi+Msi2si
X
X
Bcl2

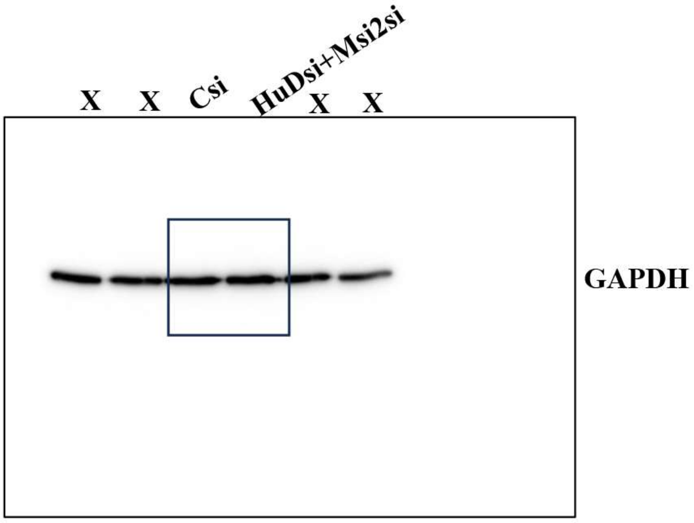
X
X
Csi
HuDsi+Msi2si
X
X
GAPDH

Supp. Fig 4

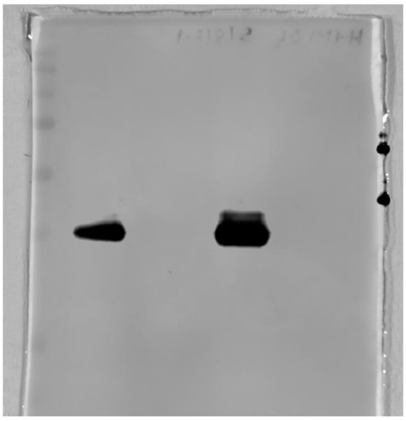

HuDA

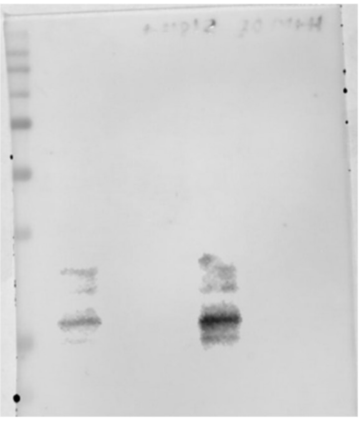

Bcl2 lower exposure

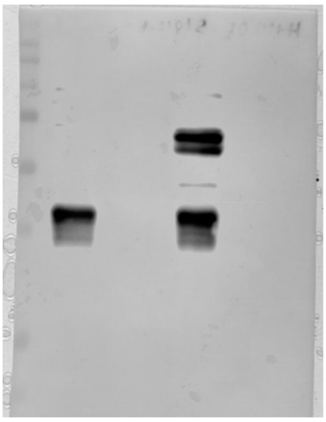

Msi2

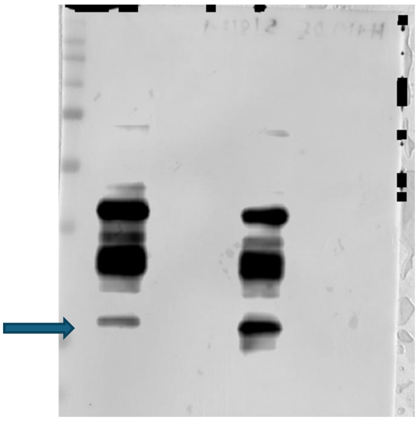

Bcl2 higher exposure

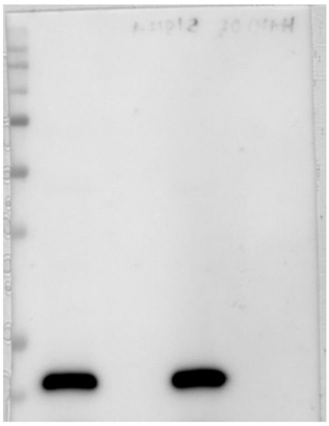

Bax

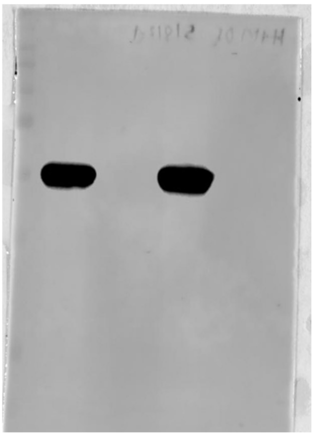

Tubulin
